# First *In Vitro*–*In Silico* Analysis for the Determination of Antimicrobial and Antioxidant Properties of 2-(4-Methoxyphenylamino)-2-oxoethyl Methacrylate and *p*-Acetamide

**DOI:** 10.1021/acsomega.3c07836

**Published:** 2024-02-09

**Authors:** Mehmet Mürşit Temüz, Nevin Çankaya, Safiye Elif Korcan, Serap Yalçin Azarkan, Tuğba Kahraman

**Affiliations:** † Department of Chemistry, Firat University, Faculty of Science, Elazığ 23119, Turkey; ‡ Vocational School of Health Services, Usak University, Usak 64200, Turkey; § Department of Medical Pharmacology, Kırsehir Ahi Evran University, Faculty of Medicine, Kırşehir 40100, Turkey; ∥ Department of Biology, Ege University, Faculty of Sciences, İzmir 35100, Turkey

## Abstract

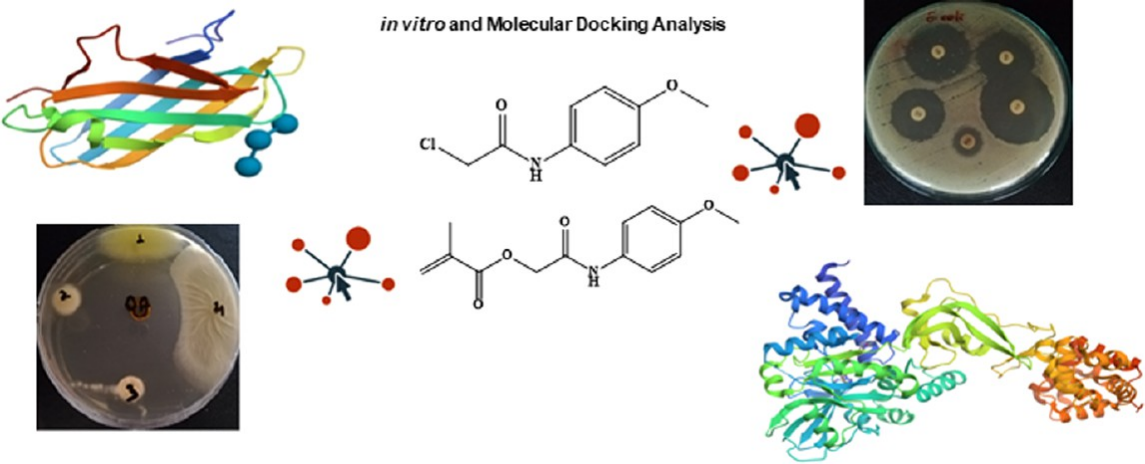

The antibacterial, antifungal, and antioxidant activities of 2-chloro-*N*-(4-methoxyphenyl)acetamide (*p*-acetamide) and 2-(4-methoxyphenylamino)-2-oxoethyl methacrylate (MPAEMA) were investigated by *in vitro* experiments and *in silico* analyses. MPAEMA has an antibacterial effect only against Gram-positive *Staphylococcus aureus*. It was determined that this did not affect any other bacteria and *Candida glabrata* yeast. On the other hand, *p*-acetamide showed antimicrobial activity against *S. aureus* ATCC 25923, *C. glabrata* ATCC 90030, *Bacillus subtilis* NRRL 744, *Enterococcus faecalis* ATCC 551289, *Escherichia coli* ATCC 25922, *Klebsiella pneumoniae* NRLLB4420, *Pseudomonas aeruginosa* ATCC 27853, and *Listeria monocytogenes* ATCC 1911. *p*-Acetamide showed the greatest antifungal effect by inhibiting the colony growth of *Trichoderma longibrachiatum* (98%). This was followed by *Mucor plumbeus* with 83% and *Fusarium solani* with 21%. MPAEMA inhibited colony growth of *T. longibrachiatum* by 95% and that of *M. plumbeus* by 91%. Also, *p*-acetamide and MPAEMA had a scavenging effect on free radicals. According to results of the *in silico* analysis, the antimicrobial effect of these compounds is due to their effect on DNA ligase. Based on drug-likeness analysis, they were found to be consistent with the Lipinski, Veber, or Ghose rule. *p*-Acetamide and MPAEMA may be used as drugs.

## 1 Introduction

Global antibiotic resistance is a very important problem in the world. Therefore, it has become a necessity to improve the use of existing drugs to design new drugs that are less susceptible to resistance development and to develop new strategies to combat resistance.^1^ Although antibiotic resistance genes have been found widely in the ecosystem, there has not been enough study on the spread of antibiotic resistance genes in plant microbiomes and the antibiotic resistance of microorganisms isolated from the ecosystem. Fungi, which have an important place in plant microbiomes, are closely related to water, soil, and air. Therefore, microorganisms in plant microbiomes can cause significant health problems in the human body.^2^

The presence of several functional groups in the therapeutic candidate chemical is crucial for drug design and development. Among the medications with amide functional groups are paracetamol, penicillin, atorvastatin, chloramphenicol, acetazolamide, trimethobenzamide, etc.^3^ Antimicrobial compounds containing the amide functional groups neutralize the antibiotic molecules by cleavage of the amide bond using hydrolases synthesized by the microorganism. It relies on a number of variables, including the amount of hydrolase enzyme produced, the specificity of the activity, and the degree of affinity for a specific antibiotic.^4^ It is also known that chlorine is used in the disinfection of water. The low cost of chlorine provides an extra advantage in its use as an antimicrobial agent.^5^ Therefore, it is possible to increase the antimicrobial effect by adding chlorine to the amide compounds. Even if the amide groups are separated from the structure of the compound by hydrolase enzymes, they can act as a chlorine carrier and cause the antimicrobial effect to continue.

The important features sought in antimicrobial compounds are low toxicity, nonmutogen, and noncarcinogenicity. Free radicals have a very unstable structure and cause oxidative stress in the cell. Antioxidants are compounds that can prevent or delay the oxidation process by neutralizing free radicals.^6^ For this reason, the fact that the compound to be used for its antimicrobial properties is also an antioxidant provides a great advantage in terms of use in living systems.^7^ Butylated hydroxyanisole was found to have antioxidant activity in the study carried out by Ibok et al.^8^

Our group used the HeLa cell line to examine the cytotoxic characteristics of 2-(4-methoxyphenylamino)-2-oxoethyl methacrylate (MPAEMA) and *p*-acetamide. The IC_50_ values for MPAEMA and *p*-acetamide were found to be 1.8 mM and 14.53 μg/mL, respectively.^9,10^ Despite this, it was discovered that MPAEMA nanocomposites made with halloysite clay (Al_2_Si_2_O_5_(OH)_4_) were not cytotoxic.^11^ Thus, the purpose of this study was to examine these two compounds’ antifungal and antibacterial qualities as their antimicrobial activities have never been studied before. While MPAEMA comprises amide and anisole functional groups, *p*-acetamide, which was employed in this work, contains chlorine, amide, and anisole functional groups. Our aims in this study are (i) to determine antioxidant capacity of this compound, (ii) to investigate antimicrobial activity against bacteria and fungi isolated from plant microbiota, and (iii) to perform molecular docking analysis of compounds to explain the antimicrobial mechanism.

## 2 Materials and Methods

**Figure 1:**
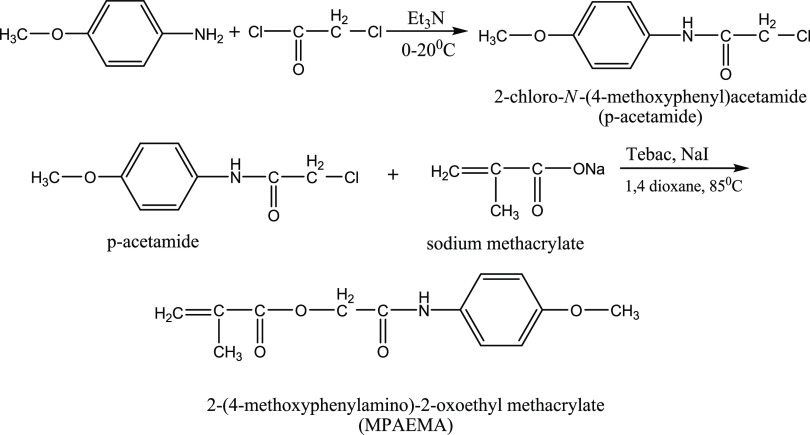
Synthesis of *p*-acetamide and MPAEMA.

Our research team employed Fourier transform infrared spectroscopy (FTIR) and NMR to characterize *p*-acetamide and MPAEMA, which were then published. These materials were also utilized in this investigation (Figure 1).^12^

### 2.1 Bacteria and Fungi Used in Biological Activity

To assess the antibacterial action, the following bacteria were used*:Escherichia coli* ATCC 25922, *Klebsiella pneumoniae* NRLLB4420, *Pseudomonas aeruginosa* ATCC 27853, *Candida glabrata* ATCC 90030, *Bacillus subtilis* NRRL 744, *Enterococcus faecalis* ATCC 551289, *Staphylococcus aureus* ATCC 25923, and *Listeria monocytogenes* ATCC 1911. All of the strains were obtained from Usak University, Vocational School of Health Services.

### 2.2 Isolation and Identification of Fungi

The isolation of fungi used in the study was made from ceramic wood, waste samples, and soil samples. Samples of waste and soil have been taken from the Usak Organized Industrial Territory. Wood samples were gathered in the vicinity of the 1 Eylül Campus of Usak University. Rose Bengal Agar (RBA) and Potato Dextrose Agar (PDA) were used to inoculate the samples after they were diluted with distilled water. They were then incubated for 7 days in the dark at 27 ± 2 °C. Chosen colonies were kept in pure culture on Rose Bengal Agar at +4 °C.

The isolated fungi were described at macroscopic and microscopic levels. PDA, Czapek Agar (CA), and Malt Extract Agar (MEA) mediums were used to cultivate the fungus. Colony growth pattern, odor, color, and sporulation pattern were observed in macroscopic identification. Microscopic identification was made on the basis of the branching pattern of conidiophores, shape and appearance of phyalids, and shape, color, and wall characteristics of conidia from slide preparations stained with lactophenol-aniline blue.^13^

For molecular identification of fungi, they were cultured on potato dextrose agar (PDA). The EurX GeneMATRIX Plant and Fungi DNA isolation kit was used for DNA isolation from fungi. To regulate the quantity and purity of DNA recovered following DNA isolation, spectrophotometric measurement (Thermo Scientific Nanodrop 2000) was carried out. Internal Transcribe Sequences 1 and 4 (ITS1-ITS4) (18S rDNA) and NL1-NL4 (D1/D2 region of the large subunit of 28S rDNA) primers were used in the polymerase chain reaction (PCR) investigation to amplify the gene regions required for determining the species. One-step PCR was performed to amplify target regions ranging between 500 and 700 bases. Solis Biodyne (Estonia) FIREPol DNA Polymerase enzyme was used in the PCR (reaction initial cycle of 5 min at 95 °C, denaturation at 95 °C for 45 s, annealing at 57 °C for 45 s, followed by 60 s of extension at 72 °C). In the PCR product purification step, the purification process was performed using the ExoSAP-ITTM PCR Product Cleanup Reagent (ThermoFisher Scientific) purification enzyme for the single band samples obtained according to the procedures of the kit. For Sanger sequencing, ABI 3730XL Sanger sequencing device (Applied Biosystems, Foster City, CA) and BigDye Terminator v3.1 Cycle sequencing kit were used. The NL1 primer was used as some species could not be identified with the ITS primer. Contig Assembly Algorithm (CAP) was used in BioEdit software to perform the process steps.

### 2.3 Determination of Antibacterial Activity by the Disk Diffusion Method

Using 24 h bacterial cultures, bacterial solutions of 0.5 McFarland turbidity were spread on Müller Hinton Agar (MHA) with a swab. After absorbing 50 μL of test *p*-acetamide and MPAEMA samples into blank discs (Bioassays), they were placed on MHA. Discs impregnated with 50 μL of dimethyl sulfoxide (DMSO) were used as negative control and vancomycin (VA_30_), penicillin (P_10_), terramycin (TE_30_), erythromycin (E_15_), and chloramphenicol (C_30_) were used as positive control. The zone diameters around the discs were measured after 24 h of incubation at 37 °C.^14^

### 2.4 Determination of Antifungal Activity by Contact Assay

A 1 cm disk of agar was removed from the middle of Petri dishes containing PDA. 20 mL of DMSO (control) or *p*-acetamide and MPAEMA samples were transferred into these wells. The samples were taken from the outer edges of the fungal species colonies. After this step, these were inoculated on PDA plates in an equidistant way to the middle well. Six days were spent incubating plates at 20 °C in the dark. The distance of fungal hyphae from the central well was measured in millimeters.

### 2.5 Determination of Free-Radical Scavenging Effect

Mixing 300 mL each of *p*-acetamide and MPAEMA sample with 5700 mL of 2,2-diphenyl-1-pixyl-hydrazil (DPPH) solution, the mixture was incubated at 27 °C for 1 h in the dark. Absorbance was measured at 515 nm with a Shimadzu brand UV-1800 spectrophotometer.^15^ Gallic acid was used as the positive control. The following formula was used to assess the sample’s capacity to scavenge the DPPH radical
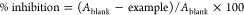


### 2.6 Molecular Docking Analyses

Molecular docking analysis is a useful tool for studying the *in silico* potential activity of molecules against proteins, DNA, etc. Predicting how tiny molecules attach to a target protein is a key step in the molecular docking process. Molecular docking software uses algorithms to search for the best possible binding mode between the ligand and the protein. The ligand’s conformation and orientation are optimized to find the lowest-energy state of the complex. The output of the molecular docking analysis provides information about the predicted binding affinity and interaction between the ligand and the protein. In this study, we investigated the interaction of *p*-acetamide and MPAEMA molecules with DNA ligase.

DNA ligases are essential enzymes that play key roles in DNA replication, recombination, and repair in all organisms.^16^ It is important to make new drug discoveries in pathogenic fungi and prokaryotic organisms. Small-molecule inhibitors of DNA ligases have long been identified using structure-based approaches.^17^ For this purpose, we studied the interaction of DNA ligase with *p*-acetamide and MPAEMA molecules by using molecular docking analysis. In this way, we examined the potential of *p*-acetamide and MPAEMA to be drug-active molecules (Table 1).

**Table 1:** SMILES Form of *p*-Acetamide and MPAEMA

molecules	SMILES form
*p*-acetamide	CICC(=O)NC1CCC(CC1)OC
MPAEMA	COC1=CC=C(NC(=O)COC(=O)C(C)=C)C=C1

The 3D structures of DNA ligase (PDB ID: 5tt5, *E. coli*) and glucoamylase (PDB ID: 4bfo, *Rhizopus oryzae*) were obtained from the Protein Data Bank (PDB) (https://www.rcsb.org/, RCSB PDB: homepage, (n.d.). https://www.rcsb.org/ (accessed July 1, 2023)). The Lipinski characteristics, including molecular weight, log *P*, and the number of hydrogen 3-bond donors and acceptors for active compounds, were identified.

The docking studies between the proteins and ligands were performed by Autodock.^18^ Autodock has been used to dock *p*-acetamide and MPAEMA with target proteins, and the chemicals formed pose conformations. The absolute energy and hydrogen bond interactions of *p*-acetamide and MPAEMA were analyzed. The Molegro Molecular Viewer tool was used to visualize the interactions between the ligands and hydrogen (H) bonds (http://molxus.io/molgro-molecular-viewer/).

### 2.7 Molecular Dynamics Analyses

The ligand–protein complex interaction was simulated using the WebGro application.^19^ A 50 ns molecular dynamics simulation was used to evaluate the ligand–protein complex’s stability. WebGro performs completely solvated molecular dynamics simulations using the GROMACS simulation program.^20−24^

## 3 Results and Discussion

### 3.1 Identification of Fungal Isolates by Conventional and Molecular Methods

**Figure 2:**
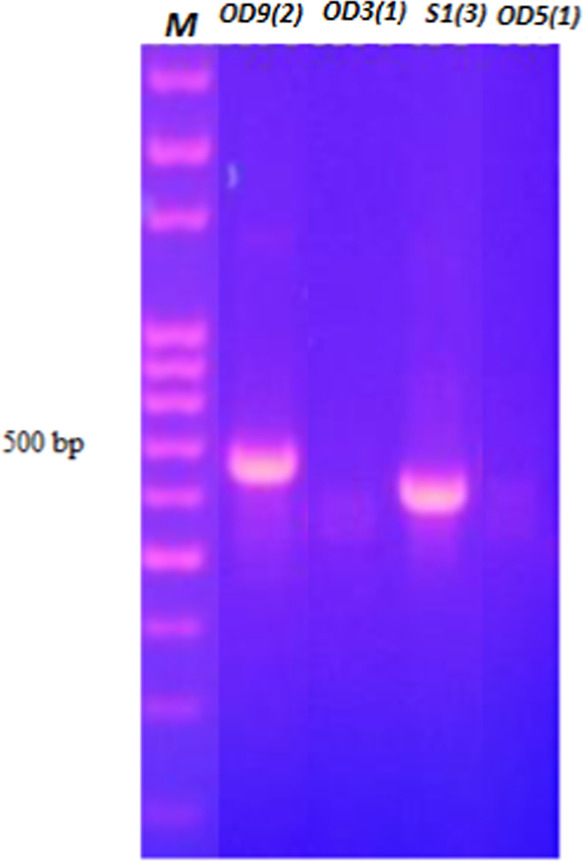
Gel image of target DNA regions of isolates varying between 500 and 700 bp.

**Figure 3:**
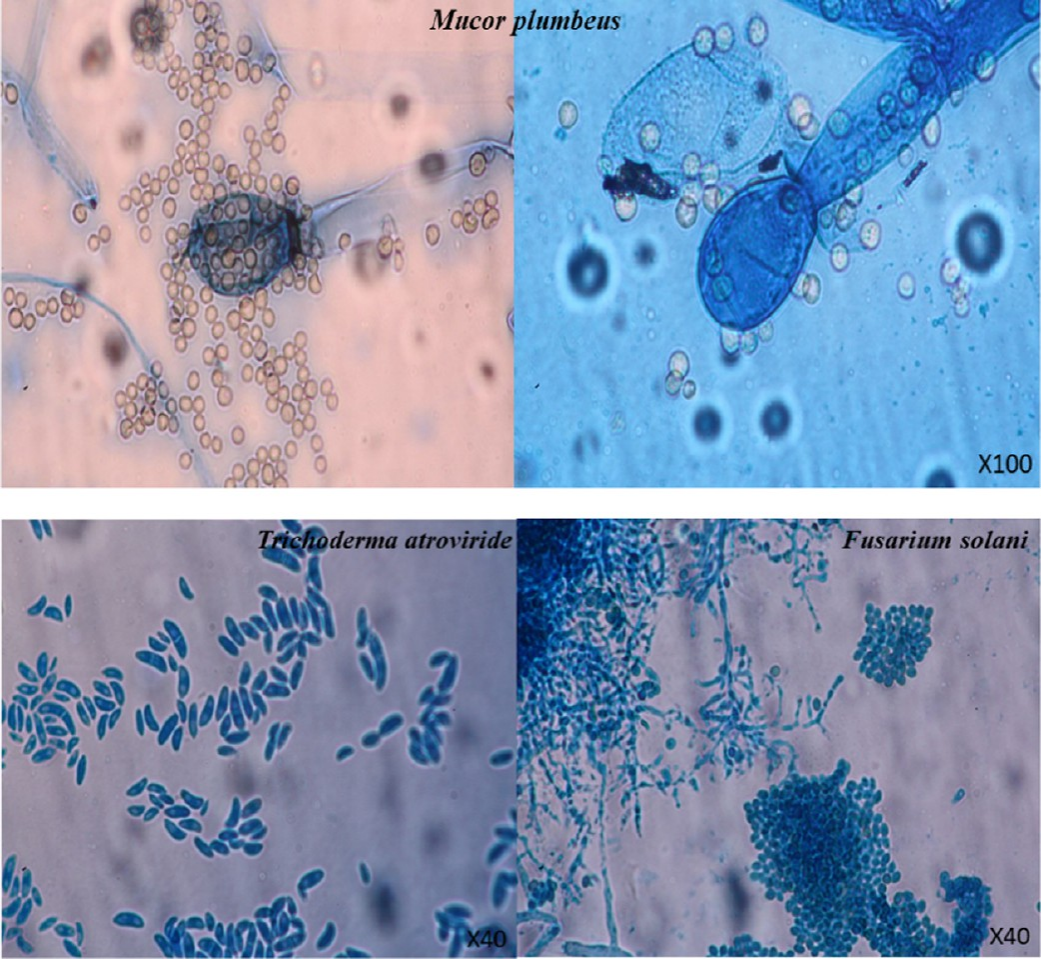
Microscopic views of fungal isolates. PDA: Potato Dextrose Agar; CA: Czapek Agar; MEA: Malt Extract Agar.

**Figure 4:**
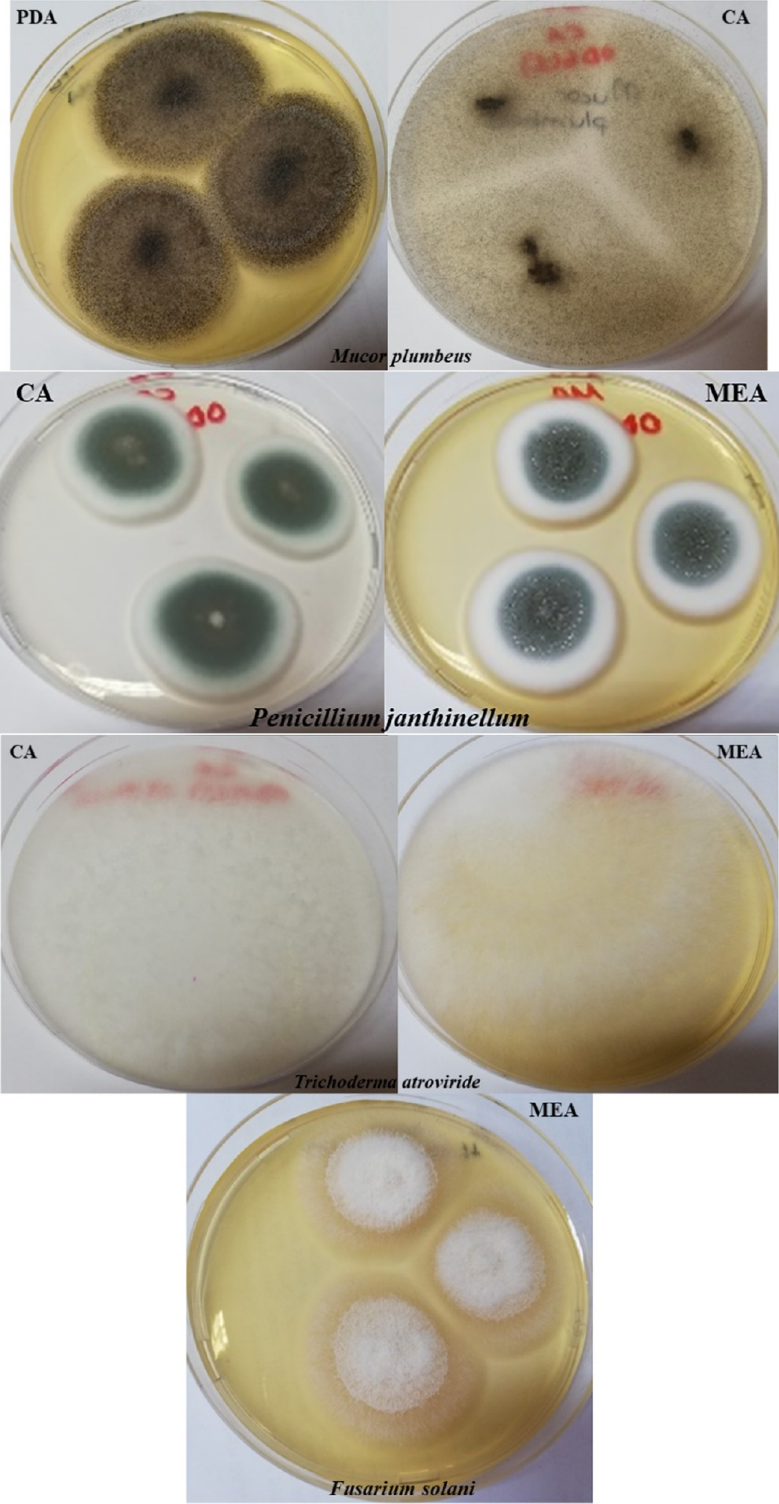
Fungal isolates on agar.

According to ITS1–ITS4 (18S rDNA) sequence analysis, two species were identified as *Penicillium janthinellum* EF634422.1 and *Fusarium solani* KT876631.1. The other two species were identified as *Mucor plumbeus* MH870585.1 (99.39%) and *Trichoderma atroviride* MH398583.1 (99.64%) based on NL1–NL4 (28S rDNA) results (Table 2 and Figure 2). Target regions varying between 500 and 700 bases obtained as a result of PCR are given in Figure 2. Classical identification of fungi was determined according to macroscopic and microscopic morphology analyses (Figures 3 and 4) 8–10. The isolate identified as *Penicillium sartoryi* according to the classical identification was found to be 100% *P. janthinellum* EF634422.1 according to the results of the molecular analysis. As a result of both molecular identification and classical identification, the other three species were identified as *M. plumbeus* MH870585.1, *F. solani* KT876631.1, and 99.64% *T. atroviride* MH398583.1 (Table 2).

**Table 2:** Results of Identification by Conventional and Molecular Methods^a^

		identification by molecular methods	
isolates	identification by conventional methods	ITS	NL
OD 3 (1)	*M. plumbeus*	ND	99.39 % *M. plumbeus* MH870585.1
OD 5 (1)	*T. atroviride*	ND	99.64 % *T. atroviride* MH398583.1
OD 9 (2)	*P. sartoryi*	100 % *P. janthinellum* EF634422.1	
S 1 (3)	*Fusarium* sp.	99.81 % *F. solani* KT876631.1	

a ND: could not be determined.

### 3.2 Determination of Antimicrobial Activity of *p*-Acetamide and MPAEMA

MPAEMA has an antibacterial effect only against Gram (+) *S. aureus*. It was determined that it did not affect any other bacteria or *C. glabrata* yeast. On the other hand, *p*-acetamide showed antimicrobial activity against Gram (+) and (−) bacteria and *C. glabrata*, except for *K. pneumoniae*. The antimicrobial effect (23 mm) of *p*-acetamide on *B. subtilis*, which is known to form spores and is resistant to adverse environmental conditions, is higher than that of all other tested microorganisms. It is seen that *p*-acetamide has a more antimicrobial effect on *B. subtilis* than vancomycin and terramycin. It is followed by *S. aureus* and *Enterococcus faecalis* with 20 mm. *p*-Acetamide also has an antimicrobial effect on *C. glabrata* (12 mm) (Table 3).

**Table 3:** Antibacterial Activity Test of *p*-Acetamide and MPAEMA^a^

	inhibition zones (mm)							
	positive control					negative control	test material	
test bacteria	VA (30)	TE (30)	C (30)	E (15)	P (10)	DMSO	*p*-acetamide	MPAEMA
*S. aureus* ATCC 25923	21	30	27	30	40		20	11
*C. glabrata*	8	19	28		7		12	
*B. subtilis*	22	16	36	29	31		23	
*E. faecalis* ATCC 551289	22	27	30	12	23		20	
*E. coli* ATCC 25922	25	12	30	26	33		8	
*K. pneumoniae* NRLLB4420	20	12	25	21	32			
*P. aeruginosa* ATCC 27853	6	9	12	6	6		10	
*L. monocytogenes* ATCC 1911	25	30	32	11	25		15	

a Vancomycin (VA_30_), penicillin (P_10_), terramycin (TE_30_), erythromycin (E_15_), and chloramphenicol (C_30_).

*p*-Acetamide carries an amide molecule as a functional group. It has been reported in the literature that molecules with an amide functional group are used as a precursor organic chemical group in the synthesis of drugs, agrochemicals, and polymers and have antifungal and antioxidant activity as well.

In this study, it can be said that the broad-spectrum antimicrobial effect of the *p*-acetamide molecule originates from the anisole functional groups. Similarly, in many studies, it has been reported that compounds having anisole and amide structures exhibit antibacterial properties against both Gram (+) and Gram (−) bacteria and *Mycobacterium tuberculosis* bacillus.^25−29^

The amide groups form hydrogen bonds with phosphatidylglycerol in the bacterial membrane and therefore the specificity of the molecule carrying the amide group against the bacterial membranes increases.^30−32^ This may facilitate the entry of small molecules, such as *p*-acetamide, which carry the amide functional group into the cell. Although MPAEMA contains amide and anisole groups, it may be more difficult to enter the cell because it has a molecular structure larger than *p*-acetamide. This may also prevent MPAEMA from having an antimicrobial effect.

Oral antifungal drugs have many undesirable side effects, especially hepatotoxicity.^33−35^ Therefore, it is of great importance to search for economical new alternative antifungal compounds with less side effects to treat fungal infections.^33,36^ Due to their reactive and chemical characteristics, amide and anisole groups play a significant functional role in pharmaceuticals.^33,37^ In our study, % fungal inhibition was calculated by comparing the fungal colony diameter in the samples using DMSO as a negative control. As seen in Table 4, *p*-acetamide showed the greatest antifungal effect by inhibiting the colony growth of *Trichoderma longibrachiatum* (98%). This was followed by *M. plumbeus* with 83% and *F. solani* with 21%. MPAEMA inhibited colony growth of *T. longibrachiatum* by 95% and colony growth of *M. plumbeus* by 91%. The antifungal effect on *P. janthinellum* was not determined. Bioactive substances with an amide group have reportedly been shown to have stronger antifungal action than the widely used medication fluconazole. Fluconazole is an antifungal agent containing [2-(2,4-difluorophenyl)-1,3-bis(1*H*-1,2,4-triazol-1-yl)-2-propanol] bis-triazole. It binds to the fungal cytochrome P-450 system, which metabolizes toxic substances taken from outside and prevents the conversion of lanosterol to ergosterol, which causes the fungal membranes to break down. Studies have shown that fluconazole has antifungal activity against Aspergillus spp., *Blastomyces dermatitidis*, Candida spp., *Coccidioides immitis*, *Cryptococcus neoformans*, *Histoplasma capsulatum*, and *Paracoccidioides brasiliensis*.^38^ The antifungal effect may result from cellular redox imbalance, disruption of the kinase signaling pathway, or inability to synthesize protein and ergosterol.^39^

**Table 4:** Antifungal Activity Test of *p*-Acetamide and MPAEMA (50 mg/mL)^a^

	colony inhibition of tested fungi (%)			
test material	*Tl*	*Fs*	*Pj*	*Mp*
*p-*acetamide	98	21	3	83
MPAEMA	95	32	NE	91

a Tl: *T. longibrachiatum*; Fs: *F. solani*; Pj: *P. janthinellum*; Mp: *M. plumbeus*; NE: not affected.

### 3.3 Antioxidant Activity Effects and Detection

The results of this investigation showed that the scavenging effects of free radicals by *p*-acetamide (99.95%) and MPAEMA (99.68%) were comparable to those of gallic acid (99.98%), which was employed as a positive control. Therefore, it can be said that *p*-acetamide and MPAEMA have a high antioxidant activity. Antioxidants are molecules that capture free radicals and prevent macromolecules in the cell from being oxidized. For this reason, studies on the use of natural and synthetic antioxidants as antioxidants are becoming increasingly important today.^16^ Amide derivatives have an important place in the fields of medicine, pharmacy, and biology not only with their antimicrobial properties but also because of their antioxidant, anesthetic, and platelet aggregation activities.^26,27^

Malki et al. investigated the antioxidant activity of five different amide derivatives (benzanilide, dodecanilide, *N*-cyclohexyloctamide, acetanilide, and acetaminophen (paracetamol)) using the DPPH, FRAP, and β-carotene linoleic acid method, and they determined that these amides showed significant antioxidant activity compared to reference antioxidants. In addition, they determined that the antioxidant effect increased with concentration.^40^

DPPH is a stable free radical that carries an unpaired electron in the nitrogen bridge. The oxidant effect of DPPH, a stable free radical, is eliminated in the presence of antioxidants that donate electrons and hydrogen atoms. The NH group in the amine may act as a hydrogen and electron donor, resulting in an antioxidant activity. In this study, it was reported that the antioxidant effect depends on the substituents on the amide and anisole groups. Anisole groups may be the cause of the ability of *p*-acetamide and MPAEMA to remove free radicals. In another study, it was stated that amides containing aromatic groups were more active than those containing aliphatic groups. However, there are no detailed studies in the literature on the alteration of biological activities by linking different functional groups at different positions.^41^

### 3.4 Results of Molecular Docking Analyses

**Figure 5:**
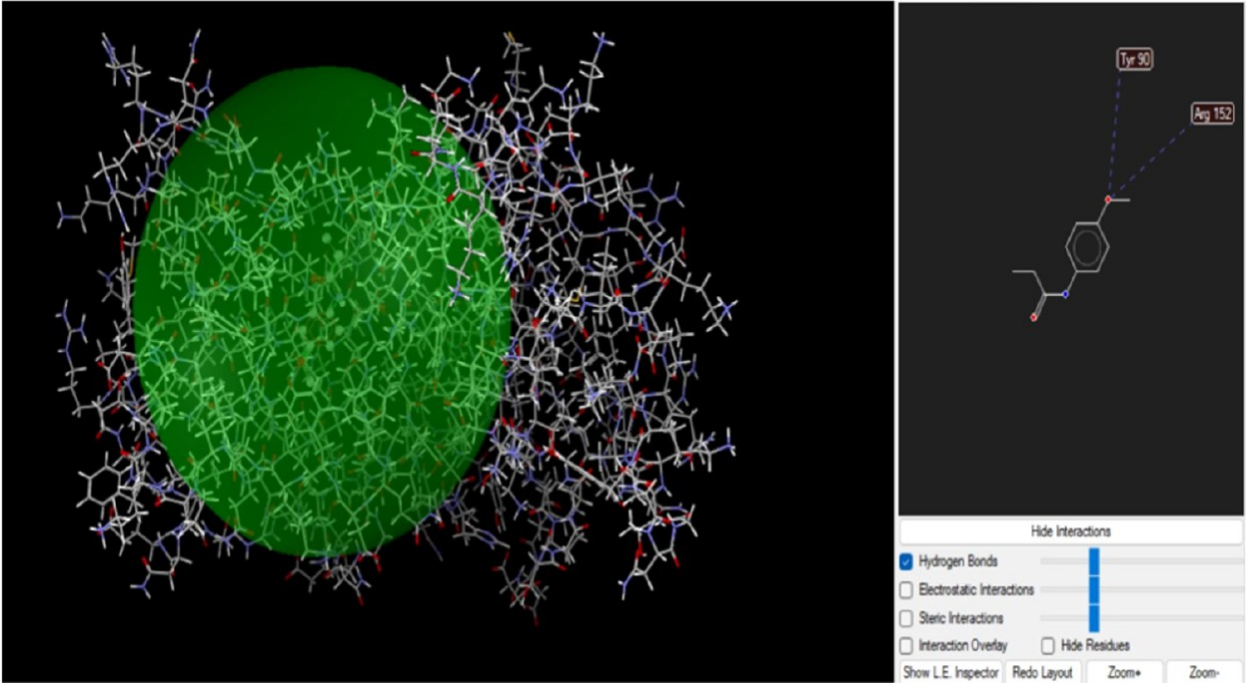
*p*-Acetamide and DNA ligase interaction.

For the best conformation of the DNA ligase to the *p*-acetamide and MPAEMA complex, binding energies of −6.91 and −7.94 kcal/mol are reported, respectively. For the best conformation of glucoamylase to the *p*-acetamide and MPAEMA complex, binding energies of −6.50 and −6.84 kcal/mol are reported, respectively. Docked complexes were visualized using discovery studio and Molegro Molecular viewer shown in Figure 5. Enzymes called DNA ligases are crucial for the repair and replication of DNA within cells.

**Figure 6:**
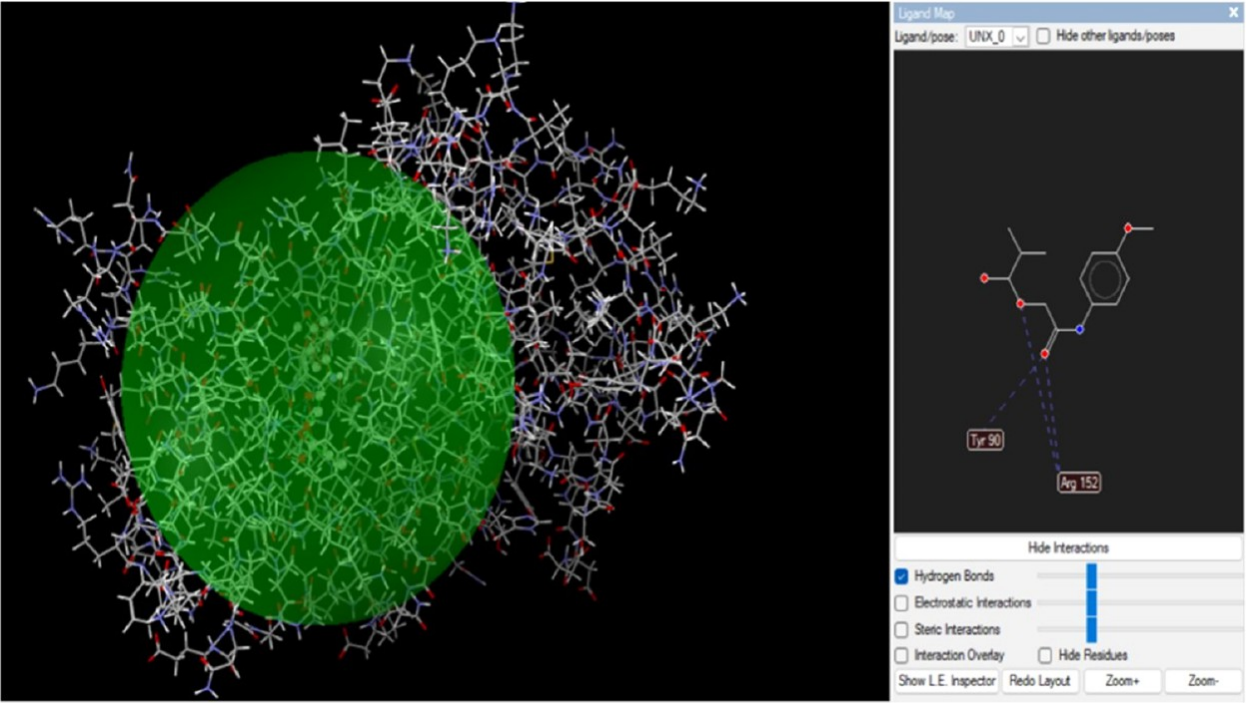
MPAEMA and DNA ligase interaction.

One crucial factor in determining a target’s affinity for a drug’s binding is the presence of hydrogen bonds. The DNA ligase protein’s Tyr 90 and Arg152 residues connect with MPAEMA three times via H-bonds (Figure 6). A hydrogen bond interaction at a distance of less than 3 Å is necessary for a strong receptor–ligand interaction. Additionally, the results revealed ligand–receptor complexes containing hydrogen bonds, all of which were located fewer than 3 Å apart. The substance binds to the DNA ligase protein with steady and high affinity. Additionally, two H-bond interactions were visible in *p*-acetamide through amino acid residues Tyr 90 and Arg152 (Figures 5 and 6).

**Figure 7:**
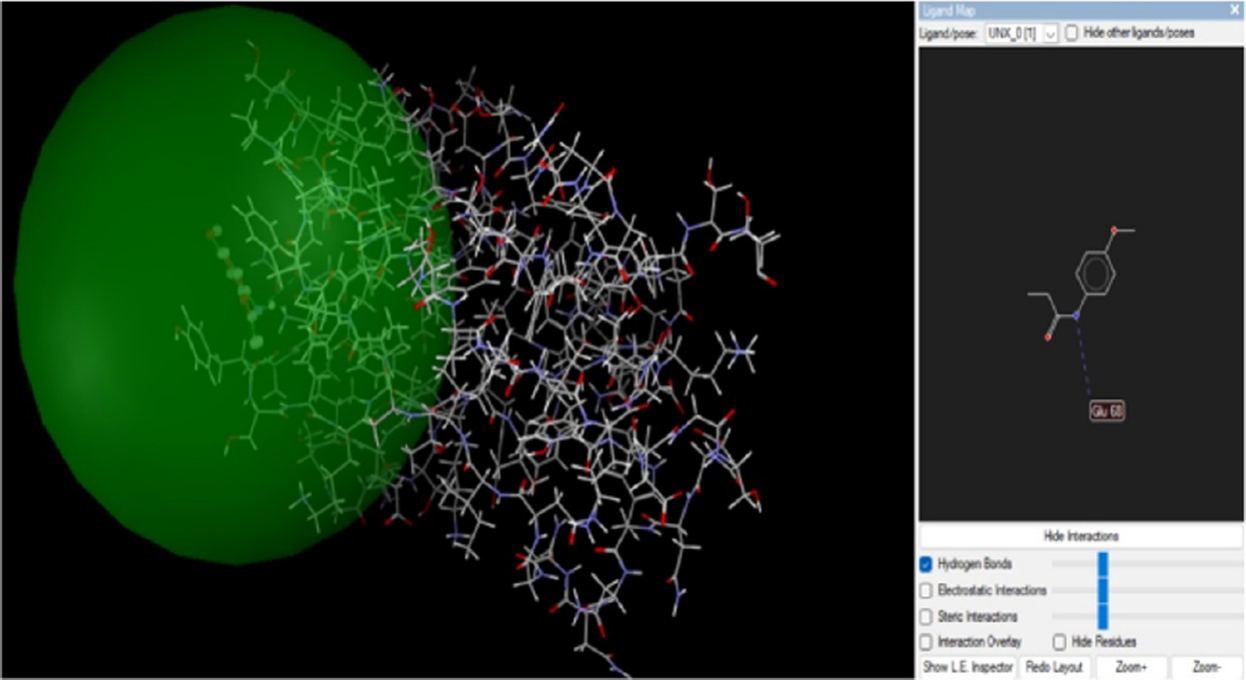
*p*-Acetamide and glucoamylase interaction.

**Figure 8:**
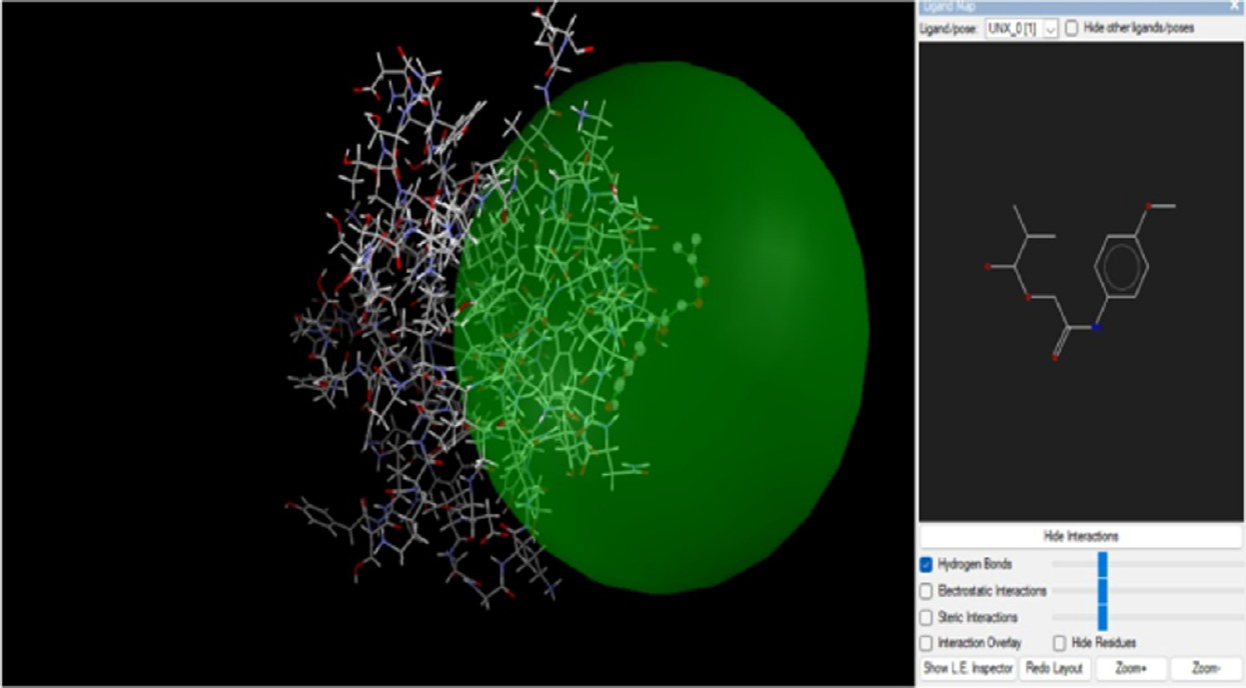
MPAEMA and glucoamylase interaction.

The hydrolysis of starch and polysaccharides into β-d-glucose is carried out by glucoamylases. *R. oryzae* glucoamylase’s catalytic C-terminal end is where glucose is found, while the starch-binding site is located at the N-terminal end. Studies have reported that glucoamylase inhibitors have antimicrobial, antifungal, and antibacterial activities and antidiabetic effects.^42−46^ Analysis results show that there is an interaction between the compounds and glucoamylase (Figures 7 and 8). H-bonds bind the Glu68 residues of the glucoamylase protein to *p*-acetamide (Figure 7).

**Figure 9:**
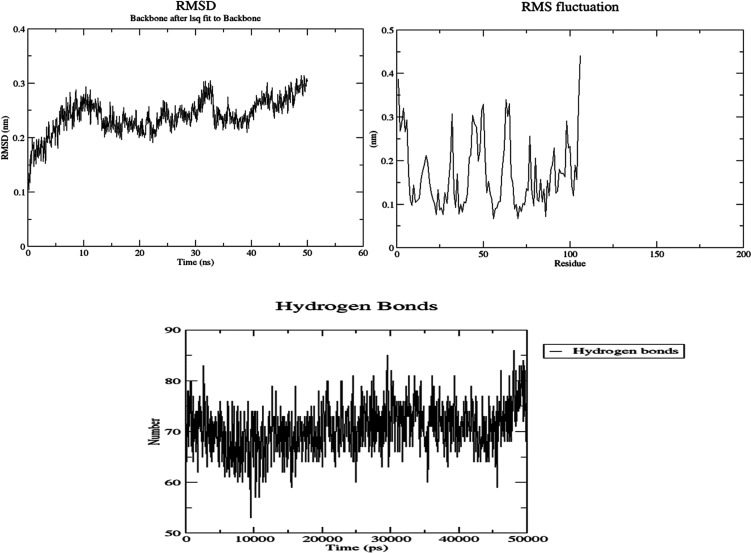
MD simulations of the stability and fluctuations of glucoamylase-*p*-acetamide (RMSD, RMSF, and hydrogen bonds).

**Figure 10:**
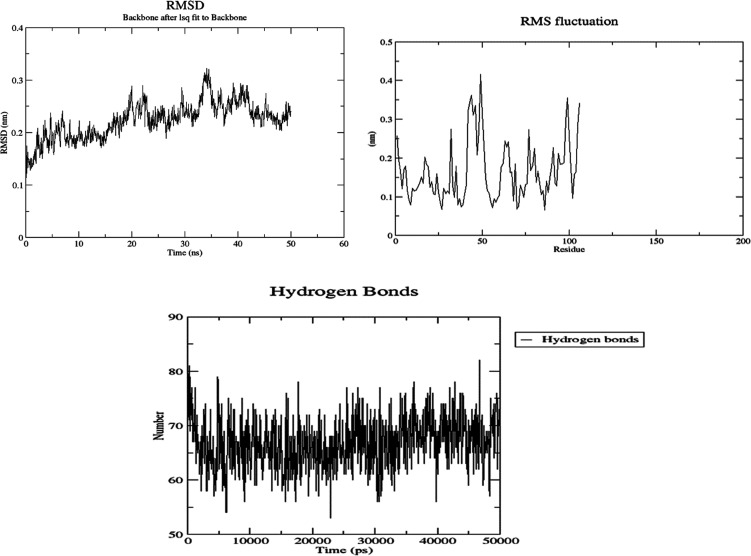
MD simulations of the stability and fluctuations of glucoamylase–MPAEMA (RMSD, RMSF, and hydrogen bonds).

**Figure 11:**
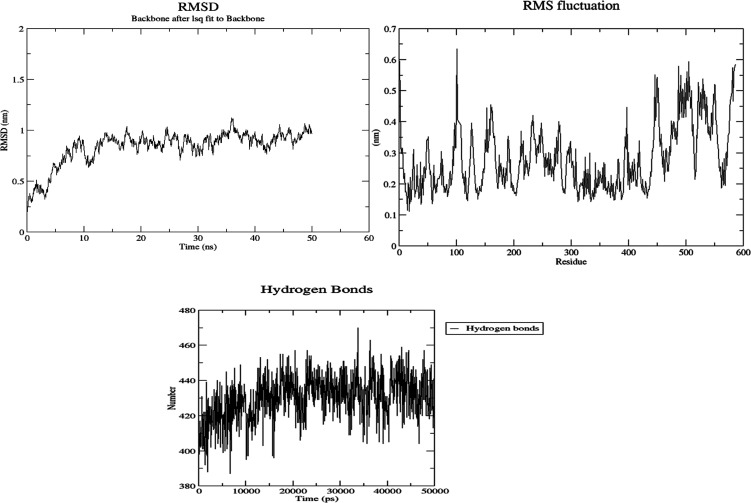
MD simulations of the stability and fluctuations of DNA ligase–*p*-acetamide (RMSD, RMSF, and hydrogen bonds).

**Figure 12:**
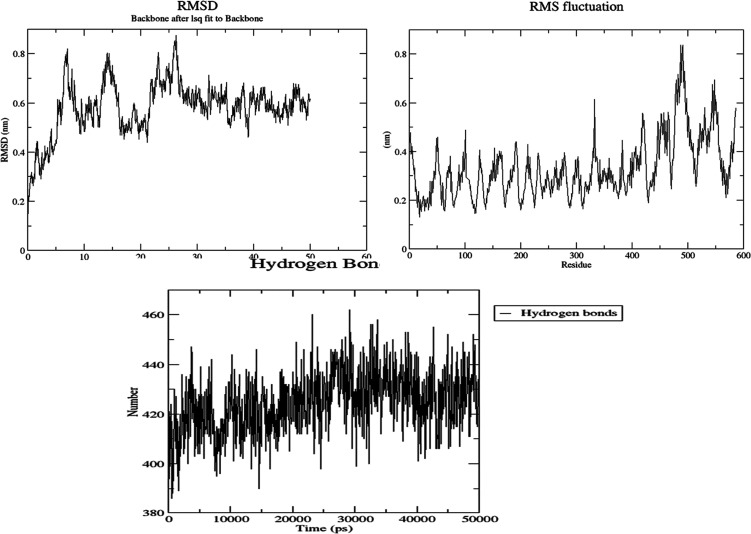
MD simulations of the stability and fluctuations of DNA ligase–MPAEMA (RMSD, RMSF, and hydrogen bonds).

Molecular dynamics (MD) simulations were used to examine the stability of *p*-acetamide and MPAEMA complexes. Each system’s dynamic trajectories were examined for stability and any changes in conformation. Tracking root-mean-square deviations (RMSD), root-mean-square fluctuations (RMSF), and hydrogen bonding patterns was all part of this investigation. All systems showed somewhat steady interaction behavior over a 50 ns time scale, according to the RMSD profiles, with the RMSD values for C-α atoms falling between 3 and 8 Å (Figures 9–12). Furthermore, time-dependent intermolecular hydrogen bond monitoring was used to evaluate the complexes’ binding properties. The complexes of DNA ligase–*p*-acetamide and DNA ligase–MPAEMA were notable for having more hydrogen bonds. The residual flexibility inside the complexes is shown by RMSF plots, where certain residues in the loops showed a larger fluctuation rate over the course of the simulation.

Computer-based ADMET analysis is currently gaining importance in drug discovery. ADMET analysis is used to determine pharmacological structures from a drug discovery perspective. SwissADME’s online tools were used to predict drug similarity and pharmacokinetics for drug candidate compounds (http://www.swissadme.ch/). Additionally, these toxicological predictions are applied to Lipinski, Ghose, and Veber rules and bioavailability scores (Table 5). Based on drug-likeness analysis, *p*-acetamide and MPAEMA were found to be consistent with the Lipinski, Veber, or Ghose rule.^27,29,47−49^*p*-Acetamide and MPAEMA may be used as drugs for other molecules.

**Table 5:** Pharmacokinetics and Drug Similarity Predictions of *p*-Acetamide and MPAEMA

	*p*-acetamide	MPAEMA
Physicochemical Properties		
formula	C_9_H_10_ClNO_2_	C_13_H_15_NO_4_
molecular weight (g/mol)	199.63	249.26
num. heavy atoms	13	18
num. atom. heavy atoms	6	6
fraction Csp3	0.22	0.23
num. rotatable bonds	4	7
num. H-bond acceptors	2	4
num. H-bond donors	1	1
molar refractivity	52.04	67.29
TPSA ( Å^2^)	38.33	64.63
Lipophilicity		
log *P*_o/w_ (iLOGP)	1.62	2.40
log *P*_o/w_ (XLOGP3)	1.65	1.95
log *P*_o/w_ (WLOGP)	1.68	1.56
log *P*_o/w_ (MLOGP)	1.54	1.34
log *P*_o/w_ (SILICOS-IT)	1.90	1.84
consensus log *P*_o/w_	1.68	1.82
Water Solubility		
log *S* (ESOL)	–2.19	–2.40
solubility	1.27 × 10 mg/mL; 6.39 × 10^–3^ mol/L	9.96 × 10^–1^ mg/mL; 3.99 × 10^–3^ mol/L
class	soluble	soluble
log *S* (Ali)	–2.07	–2.93
solubility	1.71 × 10 mg/mL; 8.54 × 10^–3^ mol/L	2.92 × 10^–1^ mg/mL; 1.17 × 10^–3^ mol/L
class	soluble	soluble
log *S* (SILICOS-IT)	–3.55	–3.46
solubility	5.61 × 10^–2^ mg/mL; 2.81 × 10^–4^ mol/L	8.68 × 10^–2^ mg/mL; 3.48 × 10^–4^ mol/L
class	soluble	soluble
Pharmacokinetics		
GI absorption	high	high
BBB permeant	yes	yes
P-gp substrate	no	no
CYP1A2 inhibitor	yes	yes
CYP2C19 inhibitor	no	no
CYP2C9 inhibitor	no	no
CYP2D6 inhibitor	no	no
CYP3A4 inhibitor	no	no
log *K*_p_ (skin permeation) (cm/s)	–6.35	–6.44
Drug-likeness		
Lipinski	yes; 0 violation	yes; 0 violation
Ghose	yes	yes
Veber	yes	yes
Egan	yes	yes
Muegge	no; 1 violation: MW < 200	yes
bioavailabilty score	0.55	0.55
Medicinal Chemistry		
PAINS	0 alert	0 alert
Brenk	1 alert: alkyl halide	1 alert: Michael acceptor-1
lead-likeness	no; 1 violation: MW < 250	no; 1 violation: MW < 250
synthetic accessibility	1.29	1.95

## 4 Conclusions

In this study, it was determined that *p*-acetamide had antibacterial and antifungal effects and MPAEMA had antifungal effects. *p*-Acetamide has a stronger antibiotic effect. The reason may be due to its smaller molecular structure compared with MPAEMA or its additional chlorine content. When drug similarities were analyzed, *p*-acetamide and MAPEMA were found to be consistent with the Lipinski, Veber, or Ghose guidelines. In conclusion, *p*-acetamide and MPAEMA may have the potential to act as drugs. *p*-Acetamide and MPAEMA can be used as drugs for other molecules. The antimicrobial effects of *p*-acetamide and MPAEMA are due to their effect on DNA ligase, which plays a role in DNA replication. We speculate that the antibacterial activity of both compounds may be due to the presence of amide groups. We may conclude that the antifungal activity of *p*-acetamide may be influenced by its interaction with glycoamylase and that its antioxidant activity may be influenced by anisole groups. In conclusion, *p*-acetamide and MPAEMA may be used as drugs. After this, *in vivo* studies and toxicology analyses of these compounds should be performed.
